# The Therapeutic Effect of Huo Xue Tong Luo Capsules in Association Research Circulation Osseous (ARCO) Stage II Osteonecrosis of the Femoral Head: A Clinical Study With an Average Follow-up Period of 7.95 Years

**DOI:** 10.3389/fphar.2021.773758

**Published:** 2021-11-23

**Authors:** Xiao-Ming He, Min-Cong He, Peng Yang, Qing-Wen Zhang, Zhen-Qiu Chen, Wei He, Qiu-Shi Wei

**Affiliations:** ^1^ Guangdong Research Institute for Orthopedics and Traumatology of Chinese Medicine, Guangzhou, China; ^2^ Joint Center, The Third Affiliated Hospital of Guangzhou University of Chinese Medicine, Guangzhou, China; ^3^ The Third Orthopaedic Region, The First Affiliated Hospital of Guangzhou University of Chinese Medicine, Guangzhou, China

**Keywords:** osteonecrosis of femoral head, ARCO stage II, therapeutic effect, risk factor, huo xue tong luo capsules

## Abstract

**Background:** Huo Xue Tong Luo (HXTL) capsules are an oral preparation that could relieve pain and ameliorate osteonecrosis in patients with asymptomatic osteonecrosis of femoral head (ONFH). We wanted to verify whether it could be a treatment option for ARCO stage II ONFH.

**Methods:** A total of 44 patients (66 hips) with ARCO stage II ONFH were recruited from June 1996 to October 2013 (clinical trial registry number: ChiCTR-RPC-15006,290). HXTL capsules were given under a specific protocol, and the endpoint was set as femoral head collapse. The clinical indicators [including visual analog scale (VAS) and Harris Hip Score (HHS)] and radiological indicators [including Tonnis classification, ARCO stage, Japanese Investigation Committee (JIC) classification, lateral preserved angle (LPA), anterior preserved angle (APA), and combined preserved angle (CPA)] before and after treatment were compared. Kaplan–Meier survival analysis and Cox regression analysis were used to identify the risk factors associated with femoral head collapse.

**Result:** Twenty-six males and 18 females with an average age of 38.3 ± 2.8 were followed for an average of 7.95 years. Forty-six of the 66 (69.7%) hips had no progression in pain or collapse, and patients exhibited a higher HHS (*p* < 0.05) after therapy. Twenty of the 66 (30.3%) hips progressed in Tonnis classification and ARCO stage, but only one of the 66 (1.5%) hips required total hip arthroplasty (THA). The Kaplan–Meier survivorship curve suggested that the survival rates were 96.97% at 5 years, 69.15% at 10 years, and 40.33% at 15 years. Patients with type A necrotic lesions on anteroposterior (AP) and frog-leg lateral (FLL) radiographs revealed 100% survival rates. Multivariate Cox regression analysis revealed that patients with an LPA ≤ 60.9 exhibited a 3.87 times higher risk of collapse of the femoral head [95% confidence interval (CI), 1.241–5.673] than did those patients with an LPA>60.9.

**Conclusion:** HXTL capsules could be a treatment option for ARCO stage II ONFH, resulting in improved hip function and delayed progression to femoral head collapse, especially when the anterior and lateral portions of the femoral head were not affected. However, an LPA of less than 60.9° may be a risk factor for collapse of the femoral head.

**Clinical Trial Registration:** http://www.chictr.org.cn/showproj.aspx?proj=10829, identifier ChiCTR-OPC-15007030

## Introduction

Osteonecrosis of femoral head (ONFH) commonly occurs in young and middle-aged adults and is associated with impaired blood supply to the femoral head secondary to the use of glucocorticoids or other factors (Cohen-Rosenblum and Cui, 2019). The literature reports that there are approximately 20 million patients with femoral head necrosis worldwide, and the number of ONFH patients in China is approximately five to seven million, increasing at a rate of 100,000–200,000 per year (Cui et al., 2016). “Necrosis–repair–collapse–osteoarthritis” is the natural pathological process that is followed by most patients with ONFH (Chen et al., 2020). There are a variety of hip preservation methods for the treatment of ONFH before collapse occurs (Mont et al., 2015). Even if there are widespread necrotic lesions, it is possible to prevent collapse. However, once collapse occurs, the treatment options are limited (Wei QS. et al., 2019; Chen et al., 2020). Studies show that Chinese herbal medicine has the potential to improve the blood flow to the necrotic femoral head, which is critical for the preservation of the hip (Wang et al., 2011; Jiang et al., 2015; Yu et al., 2016; Zhang et al., 2018). Huo Xue Tong Luo (HXTL) capsules, containing seven vasoactive herbal compounds, have been shown to delay joint collapse in patients with asymptomatic ONFH (Wei QS. et al., 2019). Because the active ingredients in HXTL capsules have been proven to promote angiogenesis, they have great potential in the treatment of ONFH (Hong et al., 2019).

Patients with ONFH tend to be young and active, and they generally have a higher rate of revision after total hip arthroplasty (THA)) which means that they are likely to have higher medical costs and experience greater physical and mental stress (Ancelin et al., 2016; Moerman et al., 2018; Kuijpers et al., 2019; Boontanapibul et al., 2020). Especially in China, due to the great social and economic pressures on young people, not all patients with ONFH can afford THA, and conservative treatment is a relatively acceptable option for them. Therefore, it is necessary to find new methods to delay the need for, or even avoid, THA (Sodhi et al., 2020). In an *in vitro* experiment examining femoral head samples, we found that the RNA level of myocardial infarction-associated transcript (Miat) in necrotic tissue was much higher than that in normal tissue and that silencing endogenous lncRNA-Miat could promote the osteogenesis of rat bone marrow mesenchymal stem cells (rBMSCs). PCR and ChIP assays showed that HXTL capsules promoted osteogenesis in rBMSCs, significantly increasing the presence of H3K27me3 and decreasing the presence of H3K4me3 in the promoter regions of Miat, demonstrating that HXTL capsules inhibited Miat expression through histone modifications. This indicates that HXTL capsules can promote osteogenesis to ameliorate ONFH by inhibiting the transcriptional expression of Miat (Fang et al., 2019). In a previous clinical trial, we used HXTL capsules to treat asymptomatic ONFH patients, and the results showed that HXTL capsules can significantly relieve pain in patients who become symptomatic and suppress necrosis progression toward femoral head collapse in the short to mid term (Wei QS. et al., 2019). Hence, we designed this clinical study to assess the therapeutic effect of HXTL capsules and reveal the risk factors related to the collapse of the femoral head to further determine whether HXTL can be an optimal treatment for ARCO stage II ONFH.

## Materials and Methods

### Inclusion and Exclusion Criteria for the Clinical Study Participants

The inclusion criteria were as follows: 1) patients with ARCO stage II ONFH diagnosed by physical examination, magnetic resonance imaging (MRI), and X-ray imaging (Yoon et al., 2020) and 2) patients who had not previously received systemic treatment. The exclusion criteria were as follows: 1) patients who were allergic to HXTL capsules; 2) patients who had undergone previous hip surgery or hip injection; 3) patients with a history of hip, femoral head, or femoral neck trauma; 4) patients with severe congenital malformation of the hip joint; 5) patients with severe bone metabolic disease; 6) patients with any diseases that could affect the hip joint, including rheumatoid arthritis, ankylosing spondylitis, joint tuberculosis, and pyogenic arthritis; and 7) patients with Type C2 necrosis lesions according to the JIC classification criteria [for which surgery is often required (Kuroda et al., 2019; Yoon et al., 2020)].

### Treatment

**FIGURE 1:**
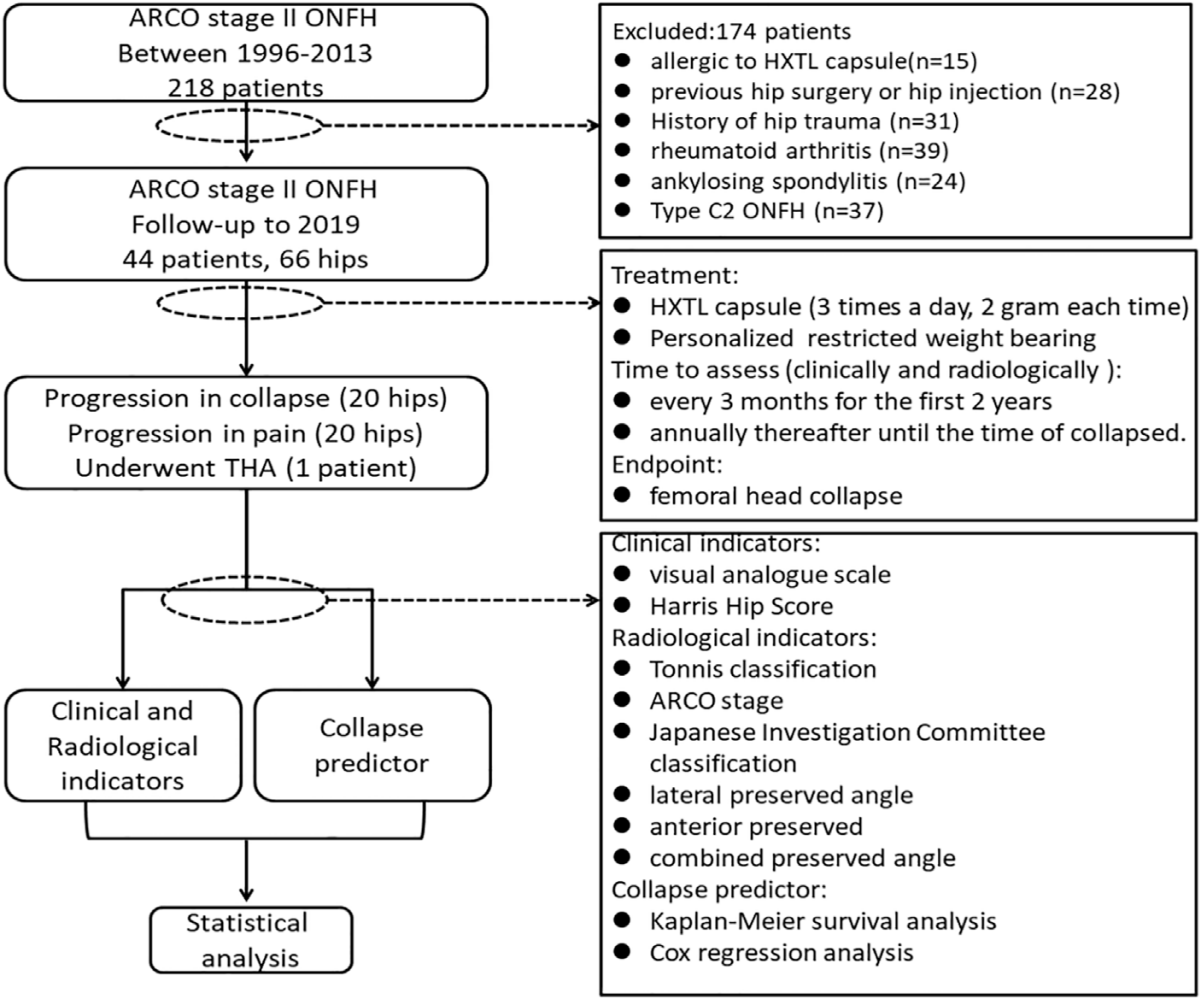
Flowchart of the study.

HXTL capsules are a vasoactive herbal preparation (institutional approval number: Z20071224) that improves the blood supply to the femoral head, promoting tissue regeneration and removing obstruction. It has been used in the clinical treatment of ONFH for more than 20 years and is made up of seven herbs, including Cajan leaf, *Angelica sinensis* (Oliv.) Diels, *Paeonia lactiflora* Pall, *Ligusticum striatum* DC, *Prunus persica* (L.) Batsch, *Carthamus tinctorius* L, and *Rehmannia glutinosa* (Gaertn.) DC (Wei QS. et al., 2019). According to the theory of traditional Chinese medicine, “obstruction leads to pain” and blood stasis can lead to local blood blockage, which is one of the causes of pain in patients with ONFH. The function of HXTL capsules is to promote qi and blood circulation, which can reduce the state of obstruction in the femoral head and thus relieve pain. Our treatment protocol was as follows: in addition to taking HXTL capsules orally three times a day (6 g/day), patients were also required to follow personalized restricted weight-bearing protocols. After bilateral hip X-ray examination in the AP and FLL positions, we assessed the location of the necrosis and bone repair, from which we developed a restricted load-bearing regimen. Asymptomatic patients with necrotic lesions not located in the weight-bearing area were allowed to walk using the normal weight-bearing areas, and regular examinations were conducted. Patients with pain and necrosis lesions that involved the weight-bearing area were advised to walk with crutches until their pain was relieved and the bone repair was complete. During follow-up, if the patient developed progressively increasing pain, it was recommended that he or she visit the hospital for further examination to determine whether the femoral head had collapsed. Disease progression has reached its endpoint when the femoral head collapses. If the femoral head did not collapse, then the patient was instructed to reduce activity and strictly follow our treatment protocol. During follow-up, all patients adjusted their treatment regimens according to the state of their bone repair. The flowchart is shown in Figure 1.

### Clinical and Radiological Evaluations

**FIGURE 2:**
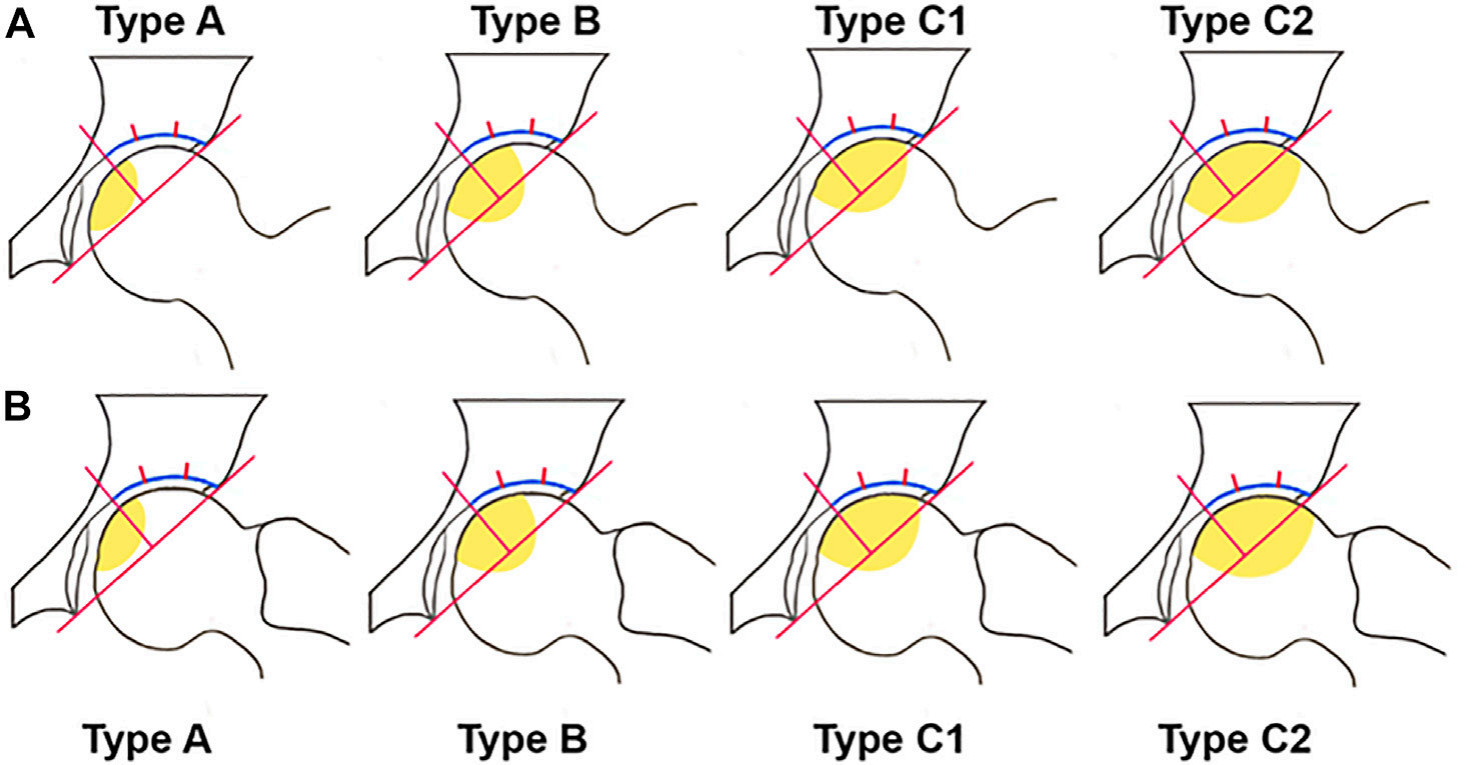
JIC classification. **(A)** JIC classification in AP view. **(B)** JIC classification in FLL view. Type A: necrotic lesion involved the medial one-third or less of the weight-bearing portion; Type B: necrotic lesion occupies the medial two-thirds or less of the weight-bearing portion; Type C1: necrotic lesion occupies more than the medial two-thirds of the weight-bearing portion but does not extend laterally to the acetabular edge; Type C2: necrotic lesion occupies more than the medial two-thirds of the weight-bearing portion and extends laterally to the acetabular edge (Sugano et al., 2002; Wei et al., 2018; Wei QS. et al., 2019).

**FIGURE 3:**
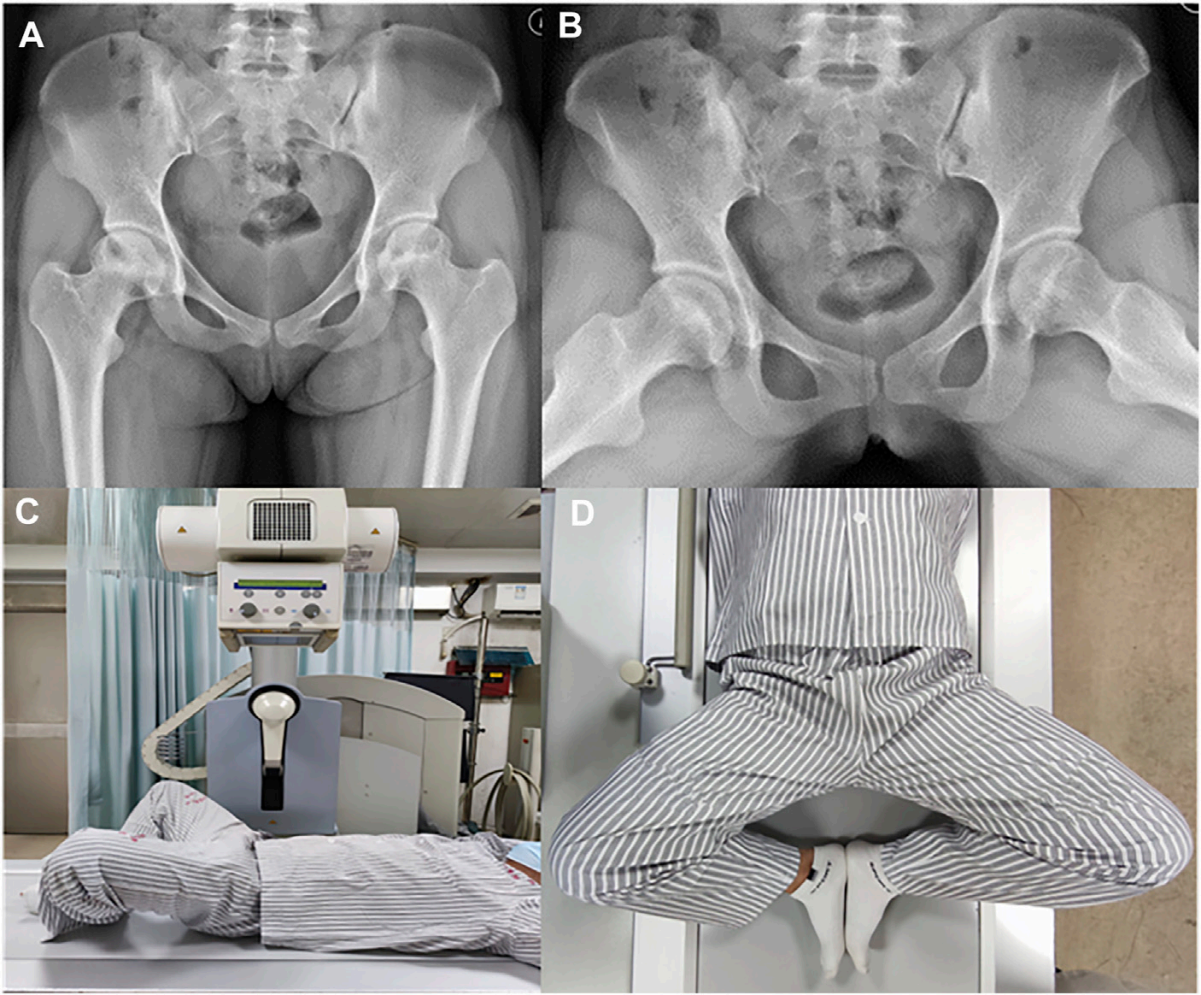
Radiological assessment **(A)** AP view of hip joint. Patients were positioned supine on the x-ray table and bilateral legs abducted in neutral position so that the distance between the two feet is equal to shoulder width. **(B)** FLL view of hip joint. **(C)** For the FLL x-rays, patients were positioned supine on the x-ray table and the bilateral hips were flexed at a degree of 30°. **(D)** For the FLL x-rays, the thigh was abducted and externally rotated while ensuring that the feet contacted close to each other at the level of ipsilateral knee. The x-ray beam was directed anterior to posterior and centered on the femoral head while the plane of the pelvis was parallel to the plane of the table (Wei QS. et al., 2019).

**FIGURE 4:**
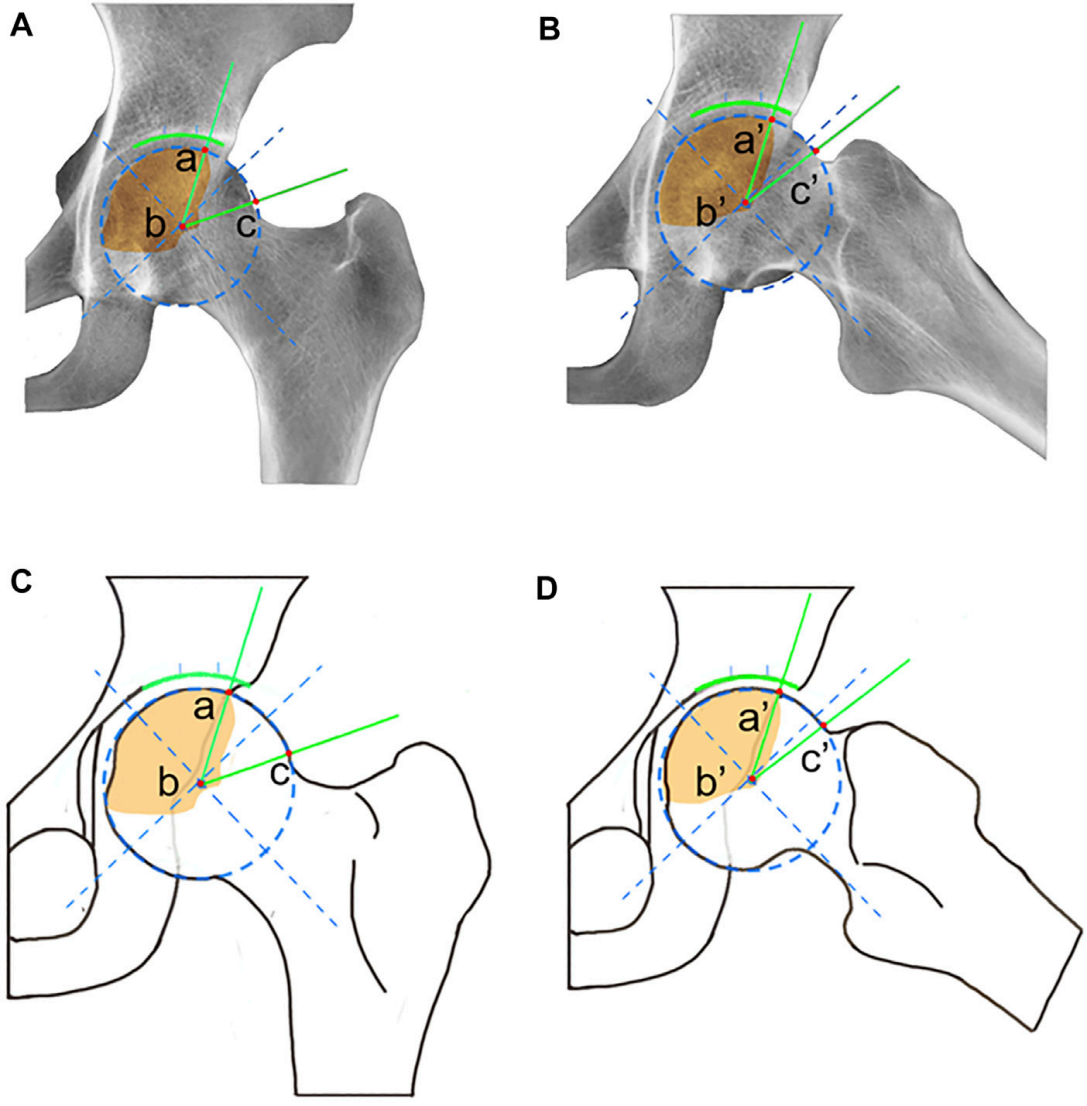
Schematic diagram of LPA, APA, and CPA. **(A)** For LPA measured in AP radiographs, point a is the intersection of necrotic area and the edge of the femoral head, point b is the center of the femoral head, and point c is the junction of the femoral head and the femoral neck. The unit of LPA is degrees, and the LPA ranges from 0 to 180°. **(B)** For APA measured in FLL radiographs, point a′ is the intersection of the necrotic area and the edge of the femoral head, point b′ is the center of the femoral head, and point c′ is the junction of the femoral head and the femoral neck. The unit of APA is degrees, and the APA ranges from 0 to 180°. CPA equal to the sum of LPA and APA.

We used the VAS and Harris Hip Score (HHS) as clinical efficacy indices to evaluate clinical function before and after treatment (Reed and Van Nostran, 2014; Hoeksma et al., 2003). The radiological evaluation included Tonnis classification, ARCO stage, JIC classification, LPA, APA, and CPA. Tonnis classification directly reflects the actual situation of hip osteoarthritis: Grade 0 means that there are no signs of osteoarthritis in hip; Grade 1 indicates slight narrowing of the joint space, slight lipping at the joint margin, and slight sclerosis of hip joint (the femoral head or acetabulum); Grade 2 indicates that small bony cysts are present on the femoral head or acetabulum, further narrowing the joint space, and that moderate flattening of the femoral head is present; Grade 3 indicates that severe deformity of the femoral head is present, with large cysts and severe narrowing of the joint space (Kovalenko et al., 2018). In the 2001 JIC classification, a type A necrotic lesion involves the medial one-third or less of the weight-bearing portion of the femoral head; a type B lesion indicates a necrotic lesion occupying the medial two-thirds or less of the weight-bearing portion; a type C lesion occupies more than the medial two-thirds of the weight-bearing portion; and type C lesions are further divided into C1 (necrotic lesions that do not extend laterally to the acetabular edge) and C2 (necrotic lesion that extends laterally to the acetabular edge) (Sugano et al., 2002). Because type C2 ONFH warrants surgery, only patients with type C1 were included in this study, and type C in this article refers only to type C1 (Kuroda et al., 2019). In addition, according to the results from our previous study, JIC classification on FLL radiographs is of clinical significance (Wei QS. et al., 2019; Wei et al., 2018). Therefore, we focused on the location of the necrotic lesions on both AP and FLL X-rays of the hip joint (Figure 2). For the AP X-rays, patients were positioned supine on the X-ray table, with both legs abducted in a neutral position so that their two feet were shoulder width apart. For the FLL X-rays, patients were positioned supine on the X-ray table, and the bilateral hips were flexed 30°. The thigh was abducted and externally rotated while ensuring that the feet contacted each other at the level of the ipsilateral knee. The X-ray beam was directed anterior to posterior and centered on the femoral head, while the plane of the pelvis was parallel to the plane of the table (Figure 3) (Wei et al., 2019b). Furthermore, to investigate the correlation between collapse and integrity of the anterior and lateral portion of the femoral head, the LPA (the larger the LPA, the smaller the necrotic lesion on the lateral portion of the femoral head) (Figure 4A, C), APA (the greater the APA, the smaller the necrotic lesion on the anterior portion of femoral head) (Figure 4B, D), and CPA (equal to the sum of LPA and APA; the larger the CPA, the smaller the necrotic lesion on the anterior and lateral portions of the femoral head) of the ONFH patients with and without collapse were measured and compared.

All patients were assessed clinically and radiologically every 3 months for the first 2 years, and annually thereafter, until the time of femoral head collapse. Each assessment was performed by two orthopedic surgeons specializing in ONFH. If the results were inconsistent, they were reviewed by two other, more senior, orthopedic surgeons with more experience, until the results were consistent.

### Statistical Analysis

Measurement data are expressed as the mean ± standard deviation. We used SPSS (version 24.0.0, SPSS Inc., Chicago, IL, USA) to analyze the data. A *p* value of less than 0.05 was considered statistically significant. We performed ROC curve analysis to explore the sensitivity and specificity of LPA, APA, and CPA in predicting femoral head collapse. Kaplan–Meier survival analysis and Cox regression analysis were used to identify the survival rate and the risk factors associated with collapse (including the location of necrosis in AP and FFL views, Tonnis classification, VAS, HHS, LPA, APA, and CPA).

## Results

A total of 44 patients with ARCO stage II ONFH were enrolled from June 1996 to October 2013, including 26 men and 18 women with an average age of 38.3 ± 2.8 years (range, 19–63 years). A total of 66 affected hips were included: 22 patients had bilateral ONFH, and 22 had unilateral ONFH. Thirty-seven patients had steroid-induced ONFH (SONFH), 20 patients had alcohol-associated ONFH (AONFH), and nine patients were diagnosed with idiopathic ONFH (IONFH) without obvious cause. According to the JIC classification criteria revised in 2019, there were 23 hips with type A, 25 hips with type B, and 18 hips with type C necrotic lesions in AP radiographs, and there were 19 hips with type A, 26 hips with type B, and 21 hips with type C necrotic lesions in FLL radiographs.

**FIGURE 5:**
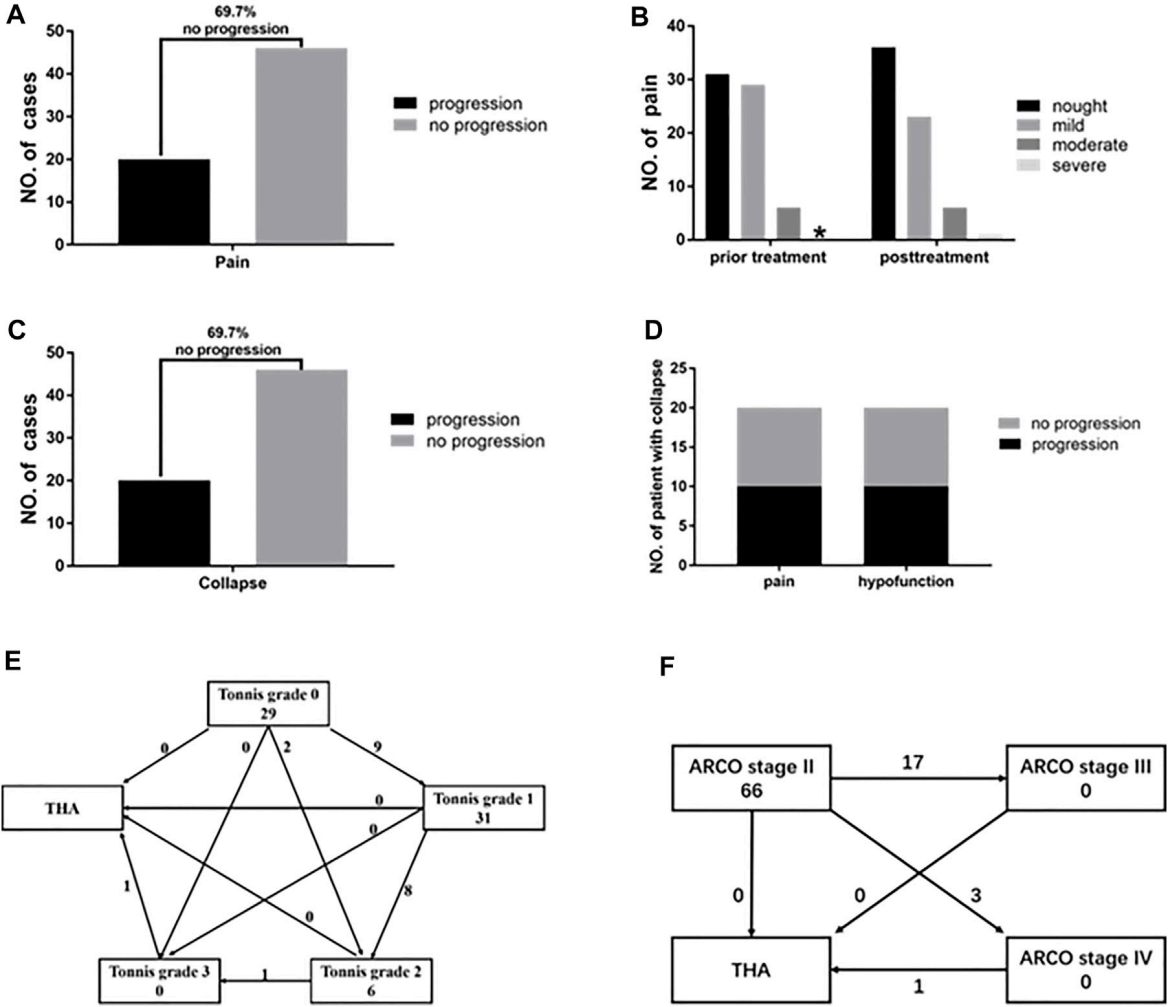
ARCO stage II ONFH patients after average 7.95 years of follow-up. **(A)** After taking HXTL capsule there were 69.7% of patients showed no progress in pain. **(B)** The degree of pain before and after taking HXTL capsule exhibited no significant difference. Nought means VAS is 0, mild means VAS from 1 to 3, moderate means VAS from 4 to 6, and severe means VAS from 7 to 10.* means the number is 0. **(C)** After taking the HXTL capsule, there were 69.7% of patients that showed no progress in collapse. **(D)** Among the 20 femoral heads that collapsed, 10 of them had no progression in pain and hypofunction (50%), and 10 of them experienced pain progression and hip dysfunction (50%). **(E)** Changes in Tonnis classification before and after treatment. **(F)** Changes in the ARCO stage of the patients after treatment.

In terms of the clinical efficacy of this study, 46 of the 66 hips (69.7%) had no progression in pain (Figure 5A), while there was also no significant difference in the degree of pain before and after treatment (Figure 5B). In terms of the radiological efficacy, 46 of the 66 femoral heads (69.7%) showed no progression toward collapse (Figure 5C). Ten of the 20 cases with collapsed femoral heads showed no worsening of pain or hip function, while 10 of the 20 experienced pain progression and increasing hip dysfunction (Figure 5D). Nine patients progressed to Tonnis grade 1 from Tonnis grade 0. Two patients progressed to Tonnis grade 2 from Tonnis grade 0, and eight patients progressed to Tonnis grade 2 from Tonnis grade 1. One patient had progressed to Tonnis grade 3 from Tonnis grade 2 and underwent THA (Figure 5E). At the end of follow-up, 17 patients had progressed to ARCO stage III, three patients developed ARCO stage IV, and one of them underwent THA (Figure 5F).

**FIGURE 6:**
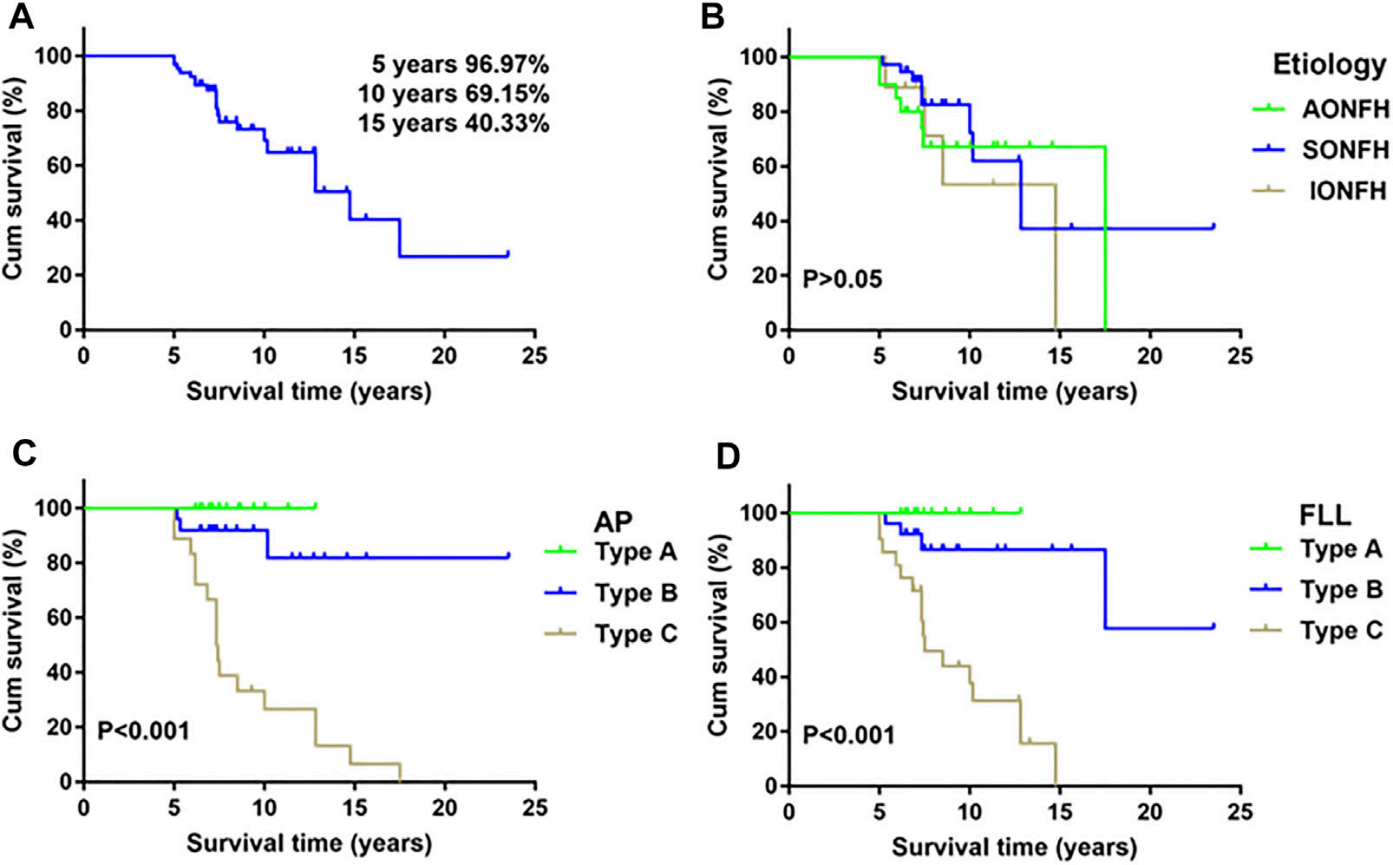
Kaplan–Meier survivorship curve. **(A)** The cumulative rates of survival (with 95% confidence intervals) are 96.97% at 5 years, 69.15% at 10 years, and 40.33% at 15 years with collapse of the femoral head as the endpoint. **(B)** Survival rates according to etiology. There were no differences (*p* = 0.56, log-rank test) in survival among etiology. **(C)** The log-rank test showed longer durations (*p* < 0.001) of survival for femoral heads with type A or type B necrotic lesions both in AP radiographs than for femoral heads with type C necrotic lesions. **(D)** The log-rank test showed longer durations (*p* < 0.001) of survival for femoral heads with type A or type B necrotic lesions both in FLL radiographs than for femoral heads with type C necrotic lesions.

The condition of the ARCO stage II ONFH patients treated with HXTL capsules was analyzed according to the etiology and location/extent of their necrotic lesions (Table 1). The survival rates of femoral heads with JIC type A in AP and FLL radiographs was 100% (Table 1). The survival rates were 96.97% at 5 years, 69.15% at 10 years, and 40.33% at 15 years with the endpoint set as collapse of the femoral head (Figure 6A). There were no differences (*p* > 0.05) among etiologies (Figure 6B). The log-rank test showed longer durations (*p* < 0.001) of survival for femoral heads with type A or type B necrotic lesions in both AP and FLL radiographs than for femoral heads with type C necrotic lesions (Figure 6C, D).

**TABLE 1:** Fate of ARCO stage II ONFH patients according to the etiology and location/extent of necrotic lesion.

Variables	Hips	Progress in pain	Progress in collapse
Total hips	66	20 (30.3%)	20 (30.3%)
Etiology			
AONFH	20 (30.3%)	2 (10.0%)	7 (35.0%)
SONFH	37 (56.1%)	15 (40.5%)	9 (56.1%)
IONFH	9 (13.6%)	3 (33.3%)	4 (13.6%)
Location			
AP			
Type A	23 (34.8%)	7 (30.4%)	0 (0)
Type B	25 (37.8%)	5 (20.1%)	3 (12.0%)
Type C	18 (27.2%)	8 (44.4%)	17 (94.4%)
FLL			
Type A	19 (28.8%)	4 (21.1%)	0 (0)
Type B	26 (39.4%)	7 (26.9%)	4 (15.4%)
Type C	21 (3.8%)	9 (42.9%)	16 (90.5%)
Extent^a^			
Small	19 (28.8%)	4 (20.0%)	1 (5.0%)
Medium	30 (45.5%)	6 (30.0%)	5 (25.0%)
Large	17 (25.8%)	10 (50.0%)	14 (70.0%)

a Small = the extent of necrosis less than 15%, medium = the extent of necrosis between 15 and 30%, large = the extent of necrosis greater than 30% (Sugano et al., 2002).

We compared various parameters between patients with collapsed femoral heads and those without collapsed femoral heads (Table 2). There were no differences (*p* > 0.05) in age, sex, follow-up period, etiology, or extent of necrotic lesions, while there were significant differences (*p* < 0.05) in the location of necrotic lesions (in AP and FLL radiographs), Tonnis classification, LPA, APA, CPA, HHS, and VAS. None of the patients with JIC type A (in AP or FLL radiographs) had femoral head collapse. Patients with intact femoral heads exhibited higher values of LPA, APA, and CPA than did patients with collapsed femoral heads. The HHS of patients with intact femoral heads was significantly higher than that of patients with collapsed femoral heads, while the VAS of patients with intact femoral heads was lower than that of patients with collapsed femoral heads (Table 2).

**TABLE 2:** Relationship between various parameters and final state of femoral head in ARCO stage II ONFH patients.

Variables	No collapsed	Collapsed	*p*
Number of hips: 66	46 (69.7%)	20 (30.3%)	-
Age (years)	35.2 ± 8.9	41.1 ± 12.7	0.069
Females: males (no.)	12:17	6:9	0.930
Follow-up period (years)	9.28 ± 3.88	8.45 ± 3.51	0.412
Etiology (alcohol: steroid: idiopathic) (no.)	13:28:5	7:9:4	0.430
Location in AP (Type A: B: C) (no.)	23:22:1	0:3:17	0.000
Location in FLL (Type A: B: C) (no.)	19:22:5	0:4:16	0.000
Extent (S: M: L) (no.)	3:25:18	1:5:14	0.066
Tonnis classification (Grade 0:1:2:3) (no.)	17:29:0:0	5:9:5:1	0.002
LPA (degree)	66.88 ± 6.75	57.48 ± 3.47	0.000
APA (degree)	66.00 ± 3.65	59.20 ± 4.52	0.000
CPA (degree)	132.88 ± 8.99	116.68 ± 7.54	0.000
HHS (point)	81.36 ± 3.06	73.45 ± 4.66	0.000
VAS (point)	0.61 ± 0.85	2.4 ± 1.66	0.000

**FIGURE 7:**
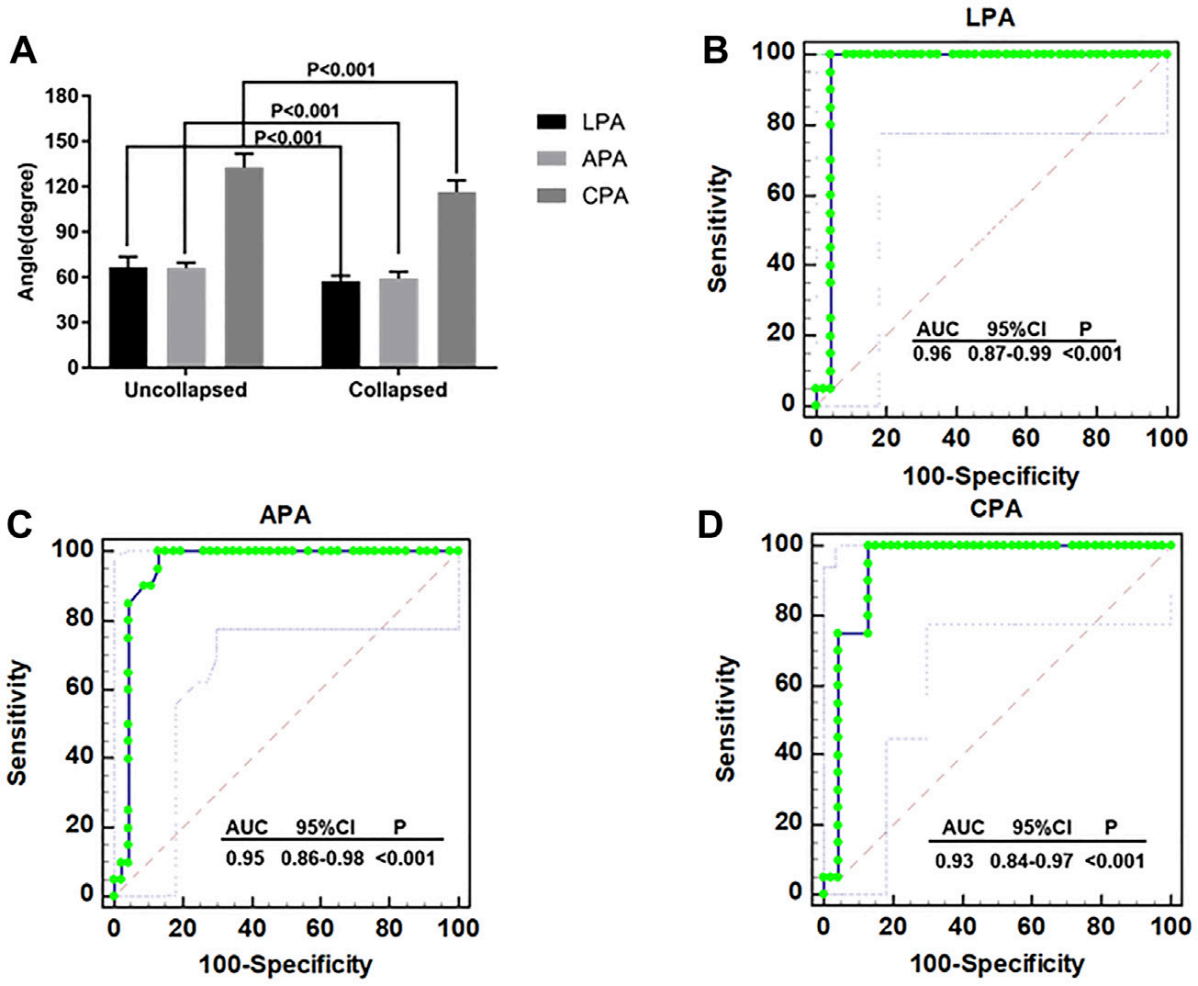
ROC curve analysis **(A)** The LPA, APA, and CPA of patients without collapse were significantly higher than patients with collapse (*p* < 0.001). **(B)** ROC curve analysis for the LPA in terms of predicting collapse (AUC = 0.96, 95% CI 0.87–0.99, *p* < 0.001, cutoff value = 60.9°, sensitivity = 100%, specificity = 95.65%). **(C)** ROC curve analysis for the APA in terms of predicting collapse (AUC = 0.95, 95% CI 0.86–0.98, *p* < 0.001, cutoff value = 62.21°, sensitivity = 100%, specificity = 86.96%). **(D)** ROC curve analysis for the CPA in terms of predicting collapse (AUC = 0.93, 95% CI 0.84–0.97, *p* < 0.001, cutoff value = 121.11°, sensitivity = 100%, specificity = 84.78%).

Moreover, all LPA, APA, or CPA values in ONFH patients without collapsed femoral heads were significantly greater than those in patients with collapsed femoral head (*p* < 0.001) (Figure 7A). ROC curves were used to analyze the sensitivity and specificity of each angle for predicting collapse. The ROC curve analysis showed LPA (AUC = 0.96, 95% CI: 0.87–0.99, *p* < 0.001, cutoff value = 60.9°, sensitivity = 100%, specificity = 95.65%) (Figure 7B), APA (AUC = 0.95, 95% CI: 0.86–0.98, *p* < 0.001, cutoff value = 62.21°, sensitivity = 100%, specificity = 86.96%) (Figure 7C), and CPA (AUC = 0.93, 95% CI: 0.84–0.97, *p* < 0.001, cutoff value = 121.11°, sensitivity = 100%, specificity = 84.78%) (Figure 7D) had high sensitivity and specificity in predicting collapse among those patients who received HXTL capsule treatment.

Univariate Cox regression analysis revealed that patients with different JIC classifications in AP and FLL views, LPA, APA, CPA, VAS, and HHS were at different (*p* < 0.05) risks for collapse (Table 3). The multivariate Cox regression analysis revealed that LPA was an independent factor associated with femoral head collapse, and patients with LPA ≤ 60.9° exhibited a 3.87 times higher risk for collapse of the femoral head [95% confidence interval (CI), 1.241–5.673] than those with LPA >60.9°.

**TABLE 3:** Univariate Cox regression analysis of the risk factors associated with collapse.

Variables	*p*	B	HR	95% CI
**Location**				
AP	0.03			
Type C	—		—	—
Type B	0.938	−13.079	—	—
Type A	0.001	−2.147	0.117	0.034–0.400
FLL	**0.007**			
Type C	—		—	—
Type B	0.939	−12.702	—	—
Type A	0.002	−1.994	0.136	0.039–0.472
Tonnis classification	**0.185**			
Grade 0	—		—	—
Grade 1	0.405	0.455	—	—
Grade 2	0.066	1.094	—	—
LPA (degree)				
>60.9	—		—	—
≤60.9	**0.014**	5.209	182.976	2.915–11,484.47
APA (degree)				
>62.21	—		—	—
≤62.21	**0.013**	4.894	133.434	2.765–6,438.96
CPA (degree)				
>121.11	—		—	—
≤121.11	**0.014**	5.209	182.97	2.915–11,484.47
VAS (point)	**0.001**			
0	—		—	—
1–3	0.048	1.186	3.275	1.013–10.587
4–6	0.000	2.452	11.608	3.133–43.011
HHS (point)	**0.007**			
80–89	—			
70–79	0.002	3.343	28.294	3.261–245.513
<70	0.002	3.216	24.935	3.262–190.605

## Discussion

In this study, we found that treatment with HXTL capsules not only improved hip function in patients with ARCO stage II ONFH but also prevented 69.7% of hips from progressing to pain and collapse. Patients with type A necrotic lesions in AP and FLL radiographs showed 100% survival rates. LPA less than 60.9° may be a risk factor for collapse of the femoral head with HXTL capsule treatment. Currently, pharmacological agents for ARCO stage II patients remain controversial, and rigorous studies are still needed to provide guidance for a treatment algorithm. Lai et al. demonstrated that bisphosphonates could decrease the rates of disease progression and the need for arthroplasty in hips (Lai et al., 2005), but the results were less favorable when bisphosphonates were combined with other modalities (Carli et al., 2014). Statins were reported to lower bone pressure and ameliorate osteonecrosis (Kang et al., 2010). Ajmal et al. found that, among 2,881 patients who received organ transplantation and underwent long-term high-dose steroid use, there was no statistically significant difference in the occurrence of ONFH between the 127 patients who took statins compared with the 201 patients who did not take statins (Ajmal et al., 2009). The therapeutic effect of bisphosphonates or statins on patients with ARCO stage II ONFH is still unclear, and more rigorous studies are needed to confirm this hypothesis. Our previous study showed that HXTL capsules can relieve hip pain and prevent joint collapse in patients with asymptomatic ONFH (Wei QS. et al., 2019). It is necessary to further determine whether HXTL can be used as an optional treatment for ARCO stage II ONFH.

In our study, 69.7% of patients experienced no aggravation in pain or progression toward femoral head collapse after oral treatment with HXTL capsules. In terms of the clinical efficacy index, patients did not show significant progress (*p* > 0.05) in VAS, but they had a significant increase (*p* < 0.05) in HHS after treatment. The increase in HHS reflects the improvement of hip function, suggesting that HXTL capsules can improve hip function in patients with ARCO stage II ONFH (Hoeksma et al., 2003). From a radiological perspective, 20 of the 66 (30.3%) hips progressed to osteoarthritis and necrosis, but 10 hips with collapse showed no further deterioration in pain or function, indicating that the changes in clinical function in the treatment of ARCO stage II with HXTL capsules are not completely consistent with the radiological changes. Although some patients progressed in radiographic appearances, they showed no deterioration in hip function. The core of the treatment for ONFH is to promote the repair of necrosis and delay or avoid the collapse of the femoral head (Zhao et al., 2020; Mont et al., 2020). A systematic literature review described the progression of 394 of 664 (59%) untreated asymptomatic hips to symptoms or collapse (Mont et al., 2010). Hernigou et al. stated that 77% (93 of 121) of untreated asymptomatic ONFH collapsed and that all 23 (100%) Steinberg stage II patients experienced femoral head collapse at an average of 11 months following the onset of pain (Hernigou et al., 2006). A prospective, randomized, open-label, multicenter study found that zoledronate did not prevent collapse of the femoral head or reduce the need for THA, while the collapse rate among Steinberg stage II patients treated with zoledronate was 57.7% (15 of 26) (Lee et al., 2015). In summary, the efficacy of HXTL capsules in patients with ARCO stage II ONFH appears to be acceptable.

**FIGURE 8:**
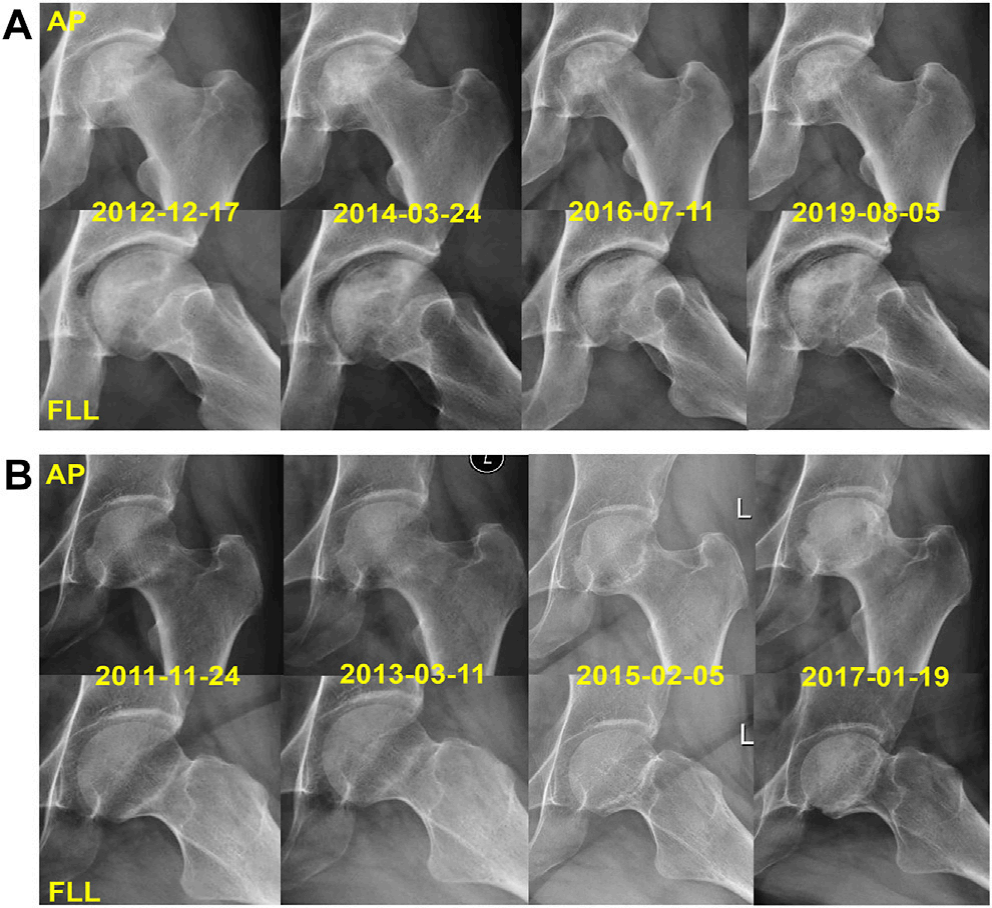
Two representative cases with JIC type C1 lesions. **(A)** X-rays of the left hip joint of a 42-year-old male with AONFH had a pretreatment LPA of 65° and APA of 67°; there was no collapse of the femoral head in the follow-up of 7.04 years. **(B)** X-rays of the left hip joint of a 38-year-old female with SONFH had a pretreatment LPA of 57.17° and APA of 59.31°; she has a femoral head collapse in 5.08 years of follow-up.

Kaplan–Meier survival analysis was used to analyze the factors that may affect femoral head collapse. The results suggested that patients with different JIC classifications on FLL or AP radiographs have different risks for collapse. Patients with type A necrotic lesions on AP and FLL radiographs had 100% survival rates. This result shows that the location of necrosis is the key factor affecting collapse when using HXTL capsules. The lateral portion of the femoral head is the key site affecting the prognosis if necrosis involves the lateral portion, and there is an increased risk of conservative treatment failure (Sugano et al., 2002; Ha et al., 2006; Gao et al., 2018; Liu et al., 2020). Combined with our results, it can be concluded that the survival rate of ARCO stage II patients treated with HXTL capsules is relatively high when necrosis does not involve the anterior or lateral portion of the femoral head. We found that the LPA, APA, and CPA of patients with collapsed femoral heads were significantly lower than those of patients without collapsed femoral heads. All three indicators had high sensitivity and specificity in predicting femoral head collapse (Figures 8A,B). According to the JIC classification and the measurement methods of LPA, APA, and CPA, we considered that these three indicators can be used as references to evaluate whether the weight-bearing area of the femoral head is involved. Although we have given the cutoff values in this paper, more studies are needed to determine whether they can be generalized to all patients with ONFH. Combined with the results of this study and the relevant literature about femoral head collapse, we performed Cox regression analysis for the factors potentially affecting collapse (Zalavras and Lieberman, 2014; Mont et al., 2020). The results suggested that LPA was an independent factor associated with collapse. Patients with an LPA ≤ 60.9° exhibited a 3.87 times higher risk of femoral head collapse [95% CI, 1.241–5.673] than those with an LPA>60.9°. Considering the sensitivity and specificity of LPA for predicting collapse, we believe that LPA can be used as an indicator to predict collapse because it can better reflect whether necrotic lesions affect the lateral portion of the femoral head, which is consistent with the study from Kuroda et al. (Kuroda et al., 2019). In summary, when the necrotic lesion did not involve the anterior or lateral portion of the femoral head, the success rate of using HXTL capsules in the treatment of ARCO stage II ONFH was relatively satisfactory.

From the perspective of modern evidence-based Chinese medicine, ischemia is the pathological state of blood stasis syndrome, which is a main feature in the pathological process of ONFH. Scholars have applied vasoactive agents to improve the revascularization of the femoral head and promote the repair of necrotic bone. Kong et al. reported that the ethyl acetate fraction of Huo Gu formula (a Chinese medicine compound that activates blood circulation) could promote the repair of necrotic bone (Kong et al., 2017). Jiang et al. found that tetramethylpyrazine can enhance vascularization and prevent osteonecrosis in rats (Jiang et al., 2015). The reparative effect of vasoactive agents on osteonecrosis can be useful and important in the treatment of ONFH. We have found that HXTL capsules can promote the repair of the femoral head (Wei QS. et al., 2019; Fang et al., 2019). In this study, we further confirmed that HXTL capsules could improve hip function in patients with ARCO stage II ONFH. In addition, we also discussed the indications for the use of HXTL capsules in the treatment of ARCO stage II ONFH. The results of this study show that HXTL capsules are effective and ideally used when neither the anterior nor the lateral portions of the femoral head are affected. In addition, LPA, APA, and CPA can be used as indicators to evaluate the anterior and lateral femoral head, which is helpful in assessing the therapeutic effect of HXTL capsules more accurately; the LPA is especially useful. Based on the above results, we will further explore the indications for HXTL capsules in the treatment of ONFH in the future.

### Limitations

First, because of the long duration of this study, it was not easy to find a matching control group. Second, although we have discussed the importance of the anterior and lateral portions of the femoral head for conservative management by LPA, APA, and CPA, whether or not they are suitable for all types of patients with ONFH needs further study. Third, the study subjects were all outpatients, and inconsistent compliance and other factors could not be excluded during the follow-up; for example, incorrect restrictive weight bearing and intense exercise or an irregular lifestyle may have influenced the study results. Moreover, the collapse rate reported in this study may be underestimated since we excluded ARCO stage II patients with JIC Type C2 lesions. Our team will carry out more studies in the future to confirm the effect of HXTL capsules on type C2 patients.

## Conclusion

In conclusion, HXTL capsules, a vasoactive herbal formula that could improve hip function and delay the progression of femoral head collapse, is an option for patients with ARCO stage II ONFH, especially when the anterior and lateral portions of the femoral head remain intact. LPA >60.9° is a risk factor for collapse of the femoral head in ARCO stage II ONFH patients treated with HXTL capsules. Multicenter prospective studies with long-term follow-up are necessary to elucidate the potential benefits of HXTL capsule treatment in ARCO stage II ONFH.

## Data Availability

The original contributions presented in the study are included in the article/supplementary material; further inquiries can be directed to the corresponding authors.
