# Corrigendum: The Therapeutic Effect of Huo Xue Tong Luo Capsules in Association Research Circulation Osseous (ARCO) Stage II Osteonecrosis of the Femoral Head: A Clinical Study With an Average Follow-up Period of 7.95 Years

**DOI:** 10.3389/fphar.2022.896418

**Published:** 2022-04-12

**Authors:** Xiao-Ming He, Min-Cong He, Peng Yang, Qing-Wen Zhang, Zhen-Qiu Chen, Wei He, Qiu-Shi Wei

**Affiliations:** ^1^ Guangdong Research Institute for Orthopedics and Traumatology of Chinese Medicine, Guangzhou, China; ^2^ Joint Center, The Third Affiliated Hospital of Guangzhou University of Chinese Medicine, Guangzhou, China; ^3^ The Third Orthopaedic Region, The First Affiliated Hospital of Guangzhou University of Chinese Medicine, Guangzhou, China

**Keywords:** osteonecrosis of femoral head, ARCO stage II, therapeutic effect, risk factor, huo xue tong luo

In the published article, there is an error in the clinical trial registry number and the URL of Clinical Trial Registration. The correct Clinical Trial Registry details are “http://www.chictr.org.cn/showproj.aspx?proj=10829, ChiCTR-OPC-15007030”.

The authors apologize for this error and state that this does not change the scientific conclusions of the article in any way. The original article has been updated.

